# Domain Satisfaction and Overall Life Satisfaction: Testing the Spillover-Crossover Model in Chilean Dual-Earner Couples

**DOI:** 10.3390/ijerph17207554

**Published:** 2020-10-17

**Authors:** Berta Schnettler, Edgardo Miranda-Zapata, Ligia Orellana, Héctor Poblete, Germán Lobos, María Lapo, Cristian Adasme-Berríos

**Affiliations:** 1 Facultad de Ciencias Agropecuarias y Forestales, Universidad de La Frontera, Temuco 478000, Chile; 2 Núcleo Científico Tecnológico en Biorecursos (BIOREN-UFRO), Universidad de La Frontera, Temuco 478000, Chile; 3 Centro de Excelencia en Psicología Económica y del Consumo, Núcleo de Ciencias Sociales, Universidad de La Frontera, Temuco 748000, Chile; edgardo.miranda@ufrontera.cl (E.M.-Z.); ligia.orellana@ufrontera.cl (L.O.); hector.poblete@ufrontera.cl (H.P.); 4 Escuela de Economía, Universidad Católica de Santiago de Guayaquil, Guayaquil 090615, Ecuador; maria.lapo@cu.ucsg.edu.ec; 5 Facultad de Economía y Negocios, Universidad de Talca, Talca 3460000, Chile; globos@utalca.cl; 6 Departamento de Economía y Administración, Universidad Católica del Maule, Talca 346000, Chile; cadasme@ucm.cl

**Keywords:** life satisfaction, job, family, food, spillover, crossover, dyadic analysis

## Abstract

The aim of this study was to examine spillover and crossover effects between job satisfaction, satisfaction with family life (SWFaL), satisfaction with food-related life (SWFoL) and overall life satisfaction (LS) in dual-earner couples. The gender of the couple members was also accounted for in these interrelationships. A sample of 473 dual-earner couples with adolescent children in Temuco, Chile, responded to a questionnaire. Both members of the couple answered the Satisfaction with Life Scale, Overall Job Satisfaction Scale, the Satisfaction with Family Life Scale and the Satisfaction with Food-related Life Scale. Using the Actor-Partner Interdependence Model and structural equation modeling, it was found that men’s LS was positively associated with their own job satisfaction, SWFaL and SWFoL (spillover), as well as with their partner’s SWFaL (crossover). Results also showed that women’s LS was positively associated with their own job satisfaction, SWFaL and SWFoL (spillover), as well as with their partner’s SWFaL and job satisfaction. Different gender patterns were found for job satisfaction and SWFoL. These findings suggest that for dual-earner couples, life satisfaction may not only be influenced by their own individual satisfaction in a life domain but also by their partner’s satisfaction in the same domain.

## 1. Introduction

As the cognitive component of subjective well-being (SWB), life satisfaction is defined as a person’s general assessment of their living conditions [1]. The life satisfaction bottom-up theoretical approach suggests that an individual’s overall life satisfaction is informed by their satisfaction in diverse life domains [2,3]. Different studies have adopted this approach to assess the influence of different life domains (e.g., work, family, health, financial situation) on overall life satisfaction at an individual level in adult samples [4,5,6,7,8]. These findings have shown that evaluations of various life domains can make independent contributions to (i.e., predict) life satisfaction [9].

The relationship between domain satisfaction and life satisfaction can be examined using the spillover model. According to this model, satisfaction in one domain positively influences other life domains as well as overall life satisfaction [10], i.e., domain satisfaction spills over into other spheres of life and on overall life satisfaction [11]. On this basis, the present study examines the contribution of three related domains—work, family and food— to overall life satisfaction. While different studies have reported positive and significant correlations between satisfaction in the job and family domains [8,12], as well as between satisfaction in the family and food domains in adult samples [13,14,15], the relationship between satisfaction in the work and food domains has not been previously assessed. However, there are studies that have demonstrated that employees with better working conditions can have family meals and cook home-made foods more frequently and have heathier diets [13,16,17].

According to the bottom-up approach, the domains that are the most immediate and important to people’s lives generally exert the strongest influence on overall life satisfaction [12]. However, bottom-up studies evaluating the contribution of various life domains to overall life satisfaction have yielded mixed results, which may be due mainly to culture-related aspects and the age of the participants [4,5,12,18]. In the US, Esterling [16] concluded that financial situation, job satisfaction and family life are the main domains that explain life satisfaction, while health was a secondary sphere. In México, Rojas [19] found that the main predictors of life satisfaction were family, economic and personal satisfaction, whereas job and health seemed less relevant. Chmiel et al. [12] concluded that only satisfaction with finances and health were significant contributors to SWB in Luxemburg, while neither satisfaction with job nor family contributed to greater SWB. Loewe et al. [6] found that satisfaction with one’s financial situation was the main predictor of overall life satisfaction in a sample of Chilean workers, followed by satisfaction with the family, work and health domains. Häusler et al. [5] found that satisfaction with one’s financial situation, followed by satisfaction with personal relationships and job satisfaction were the most important predictors of overall life satisfaction in four European Union (EU) countries. Viñas-Bardolet et al. [8] found that satisfaction with the standard of living, followed by the domains of family, social relationships and then work, were the main contributors to life satisfaction in workers in 28 EU countries. In a United States sample, Busseri and Mise [4] found that satisfaction with close relationships (such as a spouse), followed by satisfaction with finances, relationships with one’s children and job satisfaction positively contributed to higher levels of overall life satisfaction. Regarding the relative contribution of the family and food domains to overall life satisfaction, Schnettler et al. [14] found that the contribution of satisfaction with family life was slightly higher than that of satisfaction with food-related life in undergraduate students’ life satisfaction in Chile. However, when the contribution of these two domains on mothers’ and adolescents’ life satisfaction was assessed, the family domain was of higher importance than satisfaction in the food domain [20,21].

However, most of the available studies assessing the influence of satisfaction in life domains on overall life satisfaction have been conducted at an individual level, neglecting the interrelations between members of a couple, which is especially relevant in dual-earner couples. The Interdependence Theory [22] recognizes the importance of mutual influences (i.e., interdependence), while the Family Systems Theory [23] underscores the interdependence between individuals, so that individuals involved in reciprocal relationships, such as family members, can influence one another in their thoughts, emotions and behaviors. Consistent with these theories, research has shown that life satisfaction [14,24], job satisfaction [25], satisfaction with family life [14,26] and satisfaction with food-related life [14,27] are correlated between members of a couple. Therefore, both theories make it possible to hypothesize that an individual’s life satisfaction is not only influenced by their own satisfaction in different life domains but also by their partner’s satisfaction in different life domains in married or cohabiting couples, which, to the best of the authors’ knowledge, has not been previously assessed.

In line with the Interdependence Theory and the Family Systems Theory, the “spillover-crossover” model (SCM) [28] posits that experiences can be transmitted from one domain to another and to overall life satisfaction. “Spillover” is the transmission of experiences between two or more given domains; “crossover” is the transmission of experiences between domains and between individuals in close relationships [28]. Spillover involves an intra-individual transfer of experiences, while crossover is an inter-individual transmission that occurs between dyads [28,29]. The crossover process is applicable to both negative and positive experiences, with distinct dynamics [30]. Studies have reported either unidirectional crossover effects, from one partner to the other, or bidirectional effects, from one partner to the other and vice versa [31]. The bidirectional crossover requires special attention in dual-earner couples as both members of this dyad must balance work, family life and other roles, such as food-related tasks [32], and they must also fulfill different roles and tasks within their relationship [33,34]. 

Related to the above, another neglected issue in the study of domain satisfaction and overall life satisfaction is gender differences [35] in different-sex couples. The unilateral crossover effects are known as asymmetric, meaning that one couple member has an influence on the other, but this influence is not mutual [30]. Research shows that women are more influenced by their male partner than men are by their female partner, which may be due to women’s socialization to be more sensitive to their partners [36]. Nevertheless, other studies show that men’s satisfaction can also be influenced by their female partners [25,34]. In addition, when the relationship between one related variable among couples and their satisfaction with life, as well as satisfaction with different domains of life, are measured at the same time, symmetric crossover effects have been reported for some outcomes and asymmetric crossover effects for others [34,37,38].

Thus, this study contributes to the literature on satisfaction in life domains and overall life satisfaction, examining overall life satisfaction among dual-earner couples. Using couple-level data and employing dyadic data analyses, this study tests the spillover of one couple member’s satisfaction in life domains on their own life satisfaction, as well as the crossover of one couple member’s satisfaction in life domains to the other member’s life satisfaction. Therefore, on the basis of the bottom-up approach to life satisfaction, the Interdependence Theory, the Family Systems Theory and the SCM, the aims of the present study were a) to explore the spillover and crossover associations between job satisfaction, satisfaction with family life, satisfaction with food-related life and overall life satisfaction in dual-earner couples with adolescent children, and b) to explore differences between spillover and crossover effects according to the gender of the couple members. Families with adolescent children were considered for this study as adolescence has been found to be particularly challenging for parental well-being [39].

Job satisfaction is traditionally defined by how employees feel and think about their work [11], which includes measuring individuals’ emotional states, affective responses and cognitive evaluations of work [40]. Different authors have suggested that the job domain determines the level of life satisfaction, given that work is one of the most important parts of an individual’s life and takes a large share of their time [41,42]. However, Edralin [43] stressed that this relationship can result in positive and negative outcomes, which in turn may lead to elevated satisfaction levels in some individuals and stress in others. In this regard, there is evidence showing a negative relationship between the job domain and overall life satisfaction [44] and even showing a lack of relationship between them [45]. Nevertheless, consistent with the positive outcome highlighted by Edralin [43], several studies support a positive relationship between job satisfaction and overall life satisfaction at an individual level in workers in different countries [5,6,11,41,42,46,47]. 

Research based on the SCM has mostly investigated the crossover of negative experiences involving the work and health domains [30], but there is evidence in the SCM literature which leads to the expectation of crossover effects between job satisfaction and life satisfaction among members of dual-earner couples. For instance, in dual-earner couples, Sanz-Vergel and Rodríguez-Muñoz [48] found that the individual’s work enjoyment was positively related to their own daily well-being (spillover); this, in turn, was transmitted to the partner (bidirectional crossover). In addition, there is also evidence showing at least unidirectional crossover associations between work-related variables and life satisfaction in dual earner-couples. Liu and Cheung [25] found that work-to-family enrichment in women was positively associated with their husbands’ life satisfaction but not vice versa. Schnettler et al. [38] found that in men, work-life balance was positively associated with their own satisfaction with life as well as with their female partner’s life satisfaction; in women, however, their work-life balance was positively associated with their own satisfaction with life, but not with that of their male partner. 

Therefore, we pose the following hypotheses:

**H1:**  *A man’s job satisfaction is positively associated with his life satisfaction (spillover effect).*

**H2:**  *A man’s job satisfaction is positively associated with his partner’s life satisfaction (crossover effect).*

**H3:**  *A woman’s job satisfaction is positively associated with her life satisfaction (spillover effect).*

**H4:**  *A woman’s job satisfaction is positively associated with her partner’s life satisfaction (crossover effect).*

Family forms a key unit of society which plays a crucial role in the individual’s psychological and social development [49,50]. The relevant role of the family domain for well-being has been stressed by numerous studies in adult samples [4,6,7,51]. Part of this role can be assessed using the concept of family life satisfaction, the person’s assessment of their own family life based on their own subjective criteria [52]. It has been reported that having good relationships within the family and greater levels of attachment (how close family members are to each other) are beneficial for a family member’s life satisfaction [53]. In this regard, different studies using the life satisfaction bottom-up approach have supported a positive relationship between satisfaction with family life and overall life satisfaction at an individual level in different countries [6,8,18,21,41,47,54]. 

Although positive crossover from the family domain to overall life satisfaction is an under-studied field, there is evidence that at least unidirectional crossover effects between satisfaction with family life and life satisfaction in dual-earner couples can be expected. On the basis of the SCM, Chen [55] found that in Taiwan, fathers’ involvement in parenting tasks positively influences their own and the mothers’ life satisfaction, while mothers’ involvement in parenting tasks influences their own life satisfaction but not the fathers’. In addition, Schnettler et al. [56], using multivariate ordinal logit models, found that mothers’ life satisfaction was positively influenced by their own satisfaction with family life as well as by the fathers’ satisfaction with family life and vice versa, in a Chilean sample of dual-headed households. 

Therefore, we pose the following hypotheses:

**H5:**  *A man’s satisfaction with family life is positively associated with his life satisfaction (spillover effect).*

**H6:**  *A man’s satisfaction with family life is positively associated with his partner’s life satisfaction (crossover effect).*

**H7:**  *A woman’s satisfaction with family life is positively associated with her life satisfaction (spillover effect).*

**H8:**  *A woman’s satisfaction with family life is positively associated with her partner’s life satisfaction (crossover effect).*

The food domain has been relatively less studied in the literature of domain satisfaction, despite the fundamental role that food-related issues play in people’s short-term and long-term well-being [57]. An important part of an average person’s life is dedicated to investing time, energy and financial resources on food and food consumption [38,57,58]. In addition, food not only provides nourishment and sustenance but also carries cultural and symbolic meaning [59]; thus, for individuals and groups, the social value of food entails more than nutrition [38,60,61]. A person’s overall cognitive assessment of their food and eating habits (including meal planning, shopping and meal preparation) is defined as satisfaction with food-related life [58]. Satisfaction with food-related life and life satisfaction have been shown to be related in adults from different countries [62,63,64,65]. In addition, some studies based on the bottom-up approach to life satisfaction have also supported a positive relationship between satisfaction with food-related life and overall life satisfaction at an individual level in adult samples [15,21,66,67].

For crossover effects in the food domain, there is only one published study that assessed spillover and crossover effects between family members regarding satisfaction with food-related life and life satisfaction [38]. However, the evidence provided by these authors shows that at least unidirectional crossover effects between satisfaction with food-related life and life satisfaction among members of dual-earner couples is to be expected. Although no crossover effects were detected between the two parents and one adolescent child, fathers’ satisfaction with food-related life was positively associated with their own life satisfaction as well as with their partners’ (the mothers’) satisfaction with life, while the mothers’ satisfaction with food-related life was positively associated with their own life satisfaction but not with their male partners’ [38].

Therefore, we pose the following hypotheses:

**H9:**  *A man’s satisfaction with food-related life is positively associated with his life satisfaction (spillover effect).*

**H10:**  *A man’s satisfaction with food-related life is positively associated with his partner’s life satisfaction (crossover effect).*

**H11:**  *A woman’s satisfaction with food-related life is positively associated with her life satisfaction (spillover effect).*

**H12:**  *A woman’s satisfaction with food-related life is positively associated with her partner’s life satisfaction (crossover effect).*

Another important issue scarcely explored in the relationship between satisfaction in life domains and overall life satisfaction is gender differences. According to Jovanović et al. [35], the association between satisfaction in life domains and overall life satisfaction may differ between men and women, as both groups manifest different socialization practices and play different social roles. However, although it is feasible that men and women derive overall life satisfaction from different domains and sources, the scarce evidence is mixed. Diner and Fujita [68] found that social resources (such as family, friends or romantic relationships) are more strongly related to life satisfaction in women than men. Pinquart and Sorensen [69] reported that income has been more strongly associated with life satisfaction in men than in women. However, a later study concluded that the importance of social resources for overall life satisfaction did not differ between women and men in some countries [35]. In addition to the differences in women’s socialization [36], Westman [70] suggested that gender differences may be related to the distinct ways men and women react to what happens to their partner, their degree of involvement in family affairs and in traditional gender-based demands and expectations. Namely, women tend to be more involved in family activities and seem to be more responsive to situations affecting their male partners than men [26].

In summary, while some studies have found gender differences in spillover and crossover effects in couples [37,71], others report the opposite [26,72] or mixed results [73]. Two considerations are made here to begin to make sense of these inconsistent findings. First, gender role theory posits that family roles are more relevant to women’s identities, while work roles are more relevant to men’s identities [74]. The second consideration is that this study is conducted in a Latin American country, in which a traditional family structure still prevails; that is, men are positioned as the main breadwinners and women remain responsible for running the household and overseeing family issues, even if they work outside the home [56]. On this two-fold basis, we pose the following hypotheses:

**H13:**  *The spillover relationship between men’s job satisfaction and their own life satisfaction is significantly higher than the crossover association between their partners’ job satisfaction and the men’s life satisfaction.*

**H14:**  *The spillover relationship between women’s job satisfaction and their own life satisfaction is significantly lower than the crossover association between their partners’ job satisfaction and the women’s life satisfaction.*

**H15:**  *The spillover relationship between men’s satisfaction with family life and their own life satisfaction is significantly lower than the crossover association between their partners’ satisfaction with family life and the men’s life satisfaction.*

**H16:**  *The spillover relationship between women’s satisfaction with family life and their own life satisfaction is significantly higher than the crossover association between their partners’ satisfaction with family life and the women’s life satisfaction.*

**H17:**  *The spillover relationship between men’s satisfaction with food-related life and their own life satisfaction is significantly lower than the crossover association between their partner’s satisfaction with food-related life and the men’s life satisfaction.*

**H18:**  *The spillover relationship between women’s satisfaction with food-related life and their own life satisfaction is significantly higher than the crossover association between their partners’ satisfaction with food-related life and the women’s life satisfaction.*

## 2. Materials and Methods 

### 2.1. Sample and Procedure

Using non-probability sampling, 473 different-sex dual-earner couples (married or cohabiting) were recruited in Temuco, Chile (Table 1). Inclusion criteria were the couple had at least one adolescent child between 10 and 15 years of age and both mother and father had a paid job. Participants were recruited from seven schools which represented varied socioeconomical status. Trained interviewers contacted parents and provided information about the study’s objectives, the strict anonymity and confidentiality of the responses and the structure of the questionnaire. Those couples in which both members agreed to participate were visited in their homes by the interviewers between August and December 2020. After the parents signed the informed consent form, interviewers personally administered the questionnaires separately to each parent, recording their responses in a QuestionPro (QuestionPro Inc) questionnaire. After responding to both questionnaires, each couple received a gift card worth approximately USD 15. The Ethics Committee of the Universidad de La Frontera approved the study protocol (Protocol Number 007/2019). 

A pilot test was conducted for the surveys with 20 families, following the same recruitment method. The pilot test was deemed satisfactory; thus, no changes were made to the questionnaires or the interview procedure.

### 2.2. Measures

Satisfaction with Life Scale (SWLS): The SWLS [1] is a five-item scale with a single dimension to evaluate the participant’s overall cognitive judgments about their life (e.g., “In most ways my life is close to my ideal”). Respondents indicate their degree of agreement with each statement using a 6-point Likert scale (1: completely disagree; 6: completely agree). The Spanish version of the SWLS was used [75]. SWLS scores are obtained from the sum of the scores from the five items.

Overall Job Satisfaction Scale (OJSS). Job satisfaction was measured using the six items selected by Agho, Price and Mueller [76] (e.g., “I find real enjoyment in my job”) from the original 18-item index developed by Brayfield and Rothe [77]. The OJSS has previously shown good internal consistency in different countries [78,79,80]. Respondents indicated their degree of agreement with each statement using a 5-point Likert scale (1: completely disagree; 5: completely agree). The Spanish version of the OJSS scale was used [81]. OJSS scores are obtained from the sum of the scores from the six items.

Satisfaction with Family Life (SWFaL): Family life satisfaction is the “conscious cognitive judgment of one’s family life based on the subjective criteria of each individual” [52]. Zabriskie and McCormick [52] proposed this adaptation of the SWLS [1], replacing the word “life” in the five original items with “family life”. Respondents indicate their degree of agreement with each of the statements using a 6-point Likert scale (1: completely disagree; 6: completely agree). The Spanish version of the SWFaL was used [15].

Satisfaction with Food-related Life (SWFoL): The SWFoL [58] is a five-item scale that evaluates a person’s overall assessment of their food and eating habits (e.g., “Food and meals are positive elements”). Respondents indicate their degree of agreement with each statement using a 6-point Likert scale (1: completely disagree; 6: completely agree). The Spanish version of the SWFoL was used [75]. SWFoL scores were obtained via from the sum of the scores from the five items. 

The Spanish-language versions of the OJSS, SWLS, SWFoL and SWFaL have previously shown good internal consistency with adult samples in Chile [14,15,20,21,34].

Both members of the couple were asked about their age, type of employment and number of working hours per week. Women reported the number of family members, the number of children, the gender of the person with the highest income in the couple and the number of days that both members of the couple eat together during the week. The socioeconomic status (SES) was determined based on the total household income and its size [81]. 

### 2.3. Data Analysis

Descriptive analyses were conducted using SPSS v.23. Following Claxton, DeLuca and van Dulmen [82], a dyadic confirmatory factor analysis (CFA) was used to examine each scale used in this study in terms of their latent structure and their psychometric properties. Internal consistency was tested using the Omega coefficient [83]. Convergent validity was assessed by inspecting the standardized factor loadings of each scale (ideally > 0.5) as well as their significance and average variance extracted (AVE, values > 0.5) [83]. Discriminant validity was supported by comparing the AVE for each scale with the square of the correlation between the factorial scores of the scales [84].

**Figure 1:**
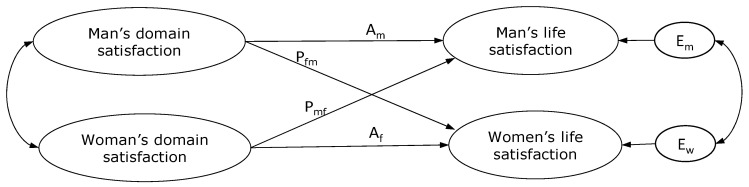
Basic actor–partner interdependence model of life domain satisfaction and life satisfaction. A_m_: Actor effect of a man’s domain satisfactions on his own life satisfaction; A_f_: actor effect of a woman’s domain satisfactions on her own life satisfaction; P_fm_: partner effect of men’s domain satisfactions on women’s life satisfaction; P_mf_: partner effect of women’s domain satisfactions on men’s life satisfaction; E_m_ and E_f_: residual errors on life satisfaction for men and women, respectively.

To test the hypotheses, structural equation modeling (SEM) [85] was used to assess the actor–partner interdependence model (APIM) with distinguishable dyads. The unit of analysis for the APIM is the dyad and the interaction between its members; each member is an actor and a partner in this analysis [85]. Therefore, it was proposed that the job satisfaction, satisfaction with family life and satisfaction with food-related life of each partner was potentially associated with both partners’ life satisfaction. “Actor effects”, or spillover, are those relationships between the job satisfaction, satisfaction with family life and satisfaction with food-related life of one member of the dyad with their own life satisfaction. “Partner effects”, or crossover, are the relationships between the levels of job satisfaction, satisfaction with family life and satisfaction with food-related life of one member of the dyad with the life satisfaction of the other member of the dyad. The APIM controls for the level of influence of one partner’s satisfaction on the other by correlating the independent variables of each dyad member (i.e., the man and woman’s job satisfaction, satisfaction with family life and satisfaction with food-related life). The APIM also allows to examine correlations between the residual errors of the dependent variables of each dyad member (i.e., the man and woman’s life satisfaction), thus controlling for other sources of interdependence between partners [85]. The basic model for spillover-crossover between domain satisfaction and overall life satisfaction is shown in Figure 1.

In modeling the fit of the data, the effects of number of children and family SES were controlled for by incorporating those variables with a direct effect on the dependent variable.

The CFA and SEM were conducted using MPlus 7.11. The parameters of the CFA and structural models were estimated via robust unweighted least squares (ULSMV). Both analyses were performed using a polychoric correlation matrix, which considered the ordinal scale of the items. The Tucker–Lewis index (TLI) and the comparative fit index (CFI) were used to determine the model fit of the data. The value of 0.90 was considered a cut-off point for establishing an acceptable fit; both the TLI and the CFI had a value above 0.95, which indicated a good fit. In addition, the root mean square error of approximation (RMSEA) was considered as a poorness-of-fit measurement. A RMSEA value lower than 0.06 indicates a good fit, while a value lower than 0.08 indicates an acceptable fit [86,87,88].

As a last step, differences between spillover (actor job satisfaction, satisfaction with family life, satisfaction with food-related life on their own life satisfaction) and crossover (partner job satisfaction, satisfaction with family life, satisfaction with food-related life on the other partner’s life satisfaction) effects were explored based on the gender of the couple members. Differences between both path coefficients were tested using a structural equation model.

## 3. Results

### 3.1. Sample Description

The sociodemographic characteristics of the sample are displayed in Table 1. This table also shows their average OJJS, SWLS, SWFoL and SWFaL scores. The mean age for women was 39.1; for men it was 42.0 years. The couples reported four family members and two children on average. Most couples corresponded to middle SES and reported that the man was the earner of the highest income. The average days per week in which both members of the couple ate together were low for the three mealtimes asked. Most women and men worked as an employee and had a 45-h work week (45 h is the legal work week in Chile). Regarding differences between couple members, men were older than women (*p* ≤ 0.001). Men had a higher average score than the women on the SWLS (*p* ≤ 0.05), SWFaL and SWFoL scales (*p* ≤ 0.001). Men were the greater proportion of workers with a 45-h work week (*p* ≤ 0.001). Women and men had similar average scores on the OJJS and similar proportions of employees and self-employed workers (*p* > 0.1).

### 3.2. Psychometric Properties of the Scales

Results for the dyadic CFAs indicated that the measurement models of OJJS (RMSEA = 0.039; CFI = 0.989; TLI = 0.984), SWFaL (RMSEA = 0.070; CFI = 0.984; TLI = 0.976), SWFoL (RMSEA = 0.051; CFI = 0.987; TLI = 0.979) and SWLS (RMSEA = 0.053; CFI = 0.990; TLI = 0.985) have good or at least acceptable fit to the data for both members of the couple. All scales showed good reliability with Omega coefficients between 0.89 and 0.99 and AVE values above 0.50. The size of factor loadings supports convergent validity, as all were statistically significant and had values above 0.5. All AVE values are greater than the square correlation between the factorial scores of the scales, which supports discriminant validity (Table 2). 

Regarding the correlations between the factorial scores of the scales, the three domains correlated positively and significantly in men and women. According to Cohen [87], the correlations between job satisfaction and satisfaction with family life (women *r* = 0.266, *p* ≤ 0.01; men *r* = 0.275, *p* ≤ 0.01) and between job satisfaction and satisfaction with food-related life (women *r* = 0.278, *p* ≤ 0.01; men *r* = 0.255, *p* ≤ 0.01) were of low strength, while the correlations between satisfaction with family life and satisfaction with food-related life (women *r* = 0.374, *p* ≤ 0.01; men *r* = 0.475, *p* ≤ 0.01) were of medium strength in both members of the couple (Table 2).

### 3.3. APIM Results

The effects of number of children and family SES were controlled for in the model that assessed the APIM. This model’s associations between both couple members’ job satisfaction, satisfaction with family life and satisfaction with food-related life, and their levels of life satisfaction, had fit indices that showed a good fit with the data (RMSEA = 0.018; CFI = 0.985; TLI = 0.983). 

**Figure 2:**
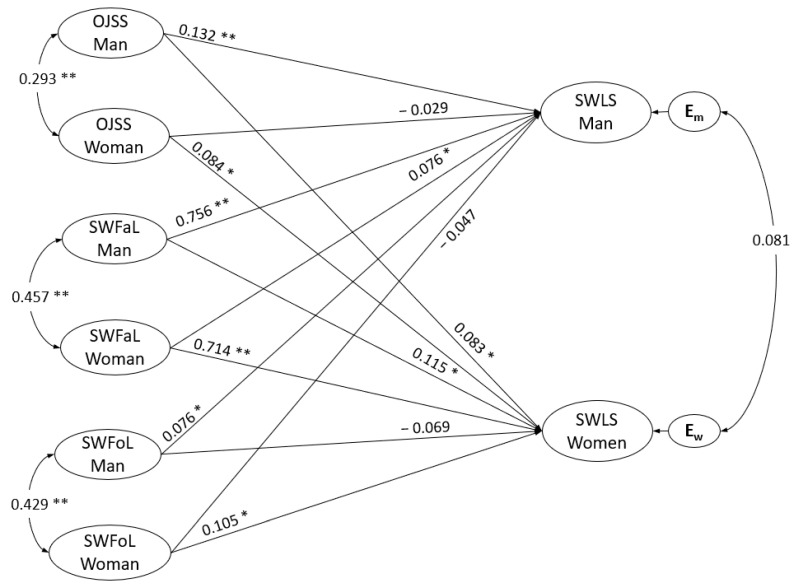
Actor–partner interdependence model of the effect of Overall Job Satisfaction Scale (OJSS), Satisfaction with Family Life (SWFaL) and Satisfaction with Food-related Life (SWFoL) on Satisfaction with Life (SWLS) in dual-earner couples. Em and Ew: residual errors on SWLS for the man and woman, respectively. * *p* < 0.05, ** *p* < 0.01. The control for the effects of number of children and family socioeconomic status on the dependent variables of the members of the couple (SWLS) was not shown in the path diagram, so as not to overload the figure.

As shown in Figure 2, significant correlations (covariances) were found for both members of the couple for job satisfaction (*r* = 0.293, *p* = 0.000), satisfaction with family life (*r* = 0.457, *p* = 0.000) and satisfaction with food-related life (*r* = 0.429, *p* = 0.000). The correlation between the residual errors of each member of the couple’s life satisfaction was not significant (*r* = 0.081, *p* = 0.243).

Figure 2 displays the results from the estimation of the structural model. Regarding job satisfaction results, the path coefficients indicated that a man’s job satisfaction was positively associated with his own life satisfaction (γ = 0.132, *p* < 0.001), thus supporting H1. The path coefficients also indicated that a man’s job satisfaction was positively associated with his female partner’s life satisfaction (γ = 0.083, *p* = 0.007), supporting H2. Likewise, the path coefficients indicated that the woman’s job satisfaction was positively associated with her own life satisfaction (γ = 0.084, *p* = 0.010), which supports H3. Path coefficients also indicated that woman’s job satisfaction was not significantly associated with her male partner’s life satisfaction (γ = −0.029, *p* = 0.429), and thus H4 was not supported.

The path coefficients for satisfaction with family life indicated that a man’s satisfaction with family life was positively associated with his own life satisfaction (γ = 0.756, *p* < 0.001), thus supporting H5, and that a man’s satisfaction with family life was positively associated with his female partner’s life satisfaction (γ = 0.115, *p* = 0.004), thus supporting H6. Likewise, the path coefficients indicated that a woman’s satisfaction with family life was positively associated with her own life satisfaction (γ = 0.714, *p* = 0.000), thus supporting H7, and that the woman’s satisfaction with family life was significantly associated with her male partner’s life satisfaction (γ = 0.076, *p* = 0.019), thus supporting H8.

The path coefficient for satisfaction with food-related life indicated that a man’s satisfaction with food-related life was positively associated with his own life satisfaction (γ = 0.076, *p* = 0.049), thus supporting H9. Path coefficients indicated that a man’s satisfaction with food-related life was not significantly associated with his female partner’s life satisfaction (γ = −0.069, *p* = 0.067), hence H10 was not supported. Likewise, the path coefficient indicated that a woman’s satisfaction with food-related life was positively associated with her own level of life satisfaction (γ = 0.105, *p* = 0.003), thus supporting H11. Path coefficients indicated that a woman’s satisfaction with food-related life was not significantly associated with her male partner’s life satisfaction (γ = −0.047, *p* = 0.241), thus not supporting H12.

The control variables did not significantly affect the model. The path coefficients for the number of children and SES on the man’s life satisfaction were 0.022 (*p* = 0.665) and −0.059 (*p* = 0.257), respectively. The path coefficients for the number of children and SES on the woman’s life satisfaction were 0.062 (*p* = 0.220) and −0.035 (*p* = 0.476), respectively. 

### 3.4. Testing Gender Differences

Results from the analysis by gender showed that the association of a man’s job satisfaction and his own life satisfaction (spillover) was significantly higher than the association of a woman’s job satisfaction and the man’s life satisfaction (crossover) (*p* < 0.001), thus supporting H13. By contrast, the association of a woman’s job satisfaction and her own life satisfaction (spillover) did not differ from the association of a man’s job satisfaction and the woman’s life satisfaction (crossover) (*p* = 0.948), thus not supporting H14.

Next, the association between satisfaction with family life and life satisfaction was tested. It was found that a man’s satisfaction with family life and his own life satisfaction (spillover) was significantly higher than the association of a woman’s satisfaction with family life and the man’s life satisfaction (crossover) (*p* < 0.001), thus not supporting H15. The association of a woman’s satisfaction with family life and her own life satisfaction (spillover) was significantly higher than the association of a man’s satisfaction with family life and the woman’s life satisfaction (crossover) (*p* < 0.001), thus supporting H16. 

Lastly, the association of a man’s satisfaction with food-related life and his own life satisfaction (spillover) did not differ from the association of a woman’s satisfaction with food-related life and the man’s life satisfaction (crossover) (*p* = 0.207), thus not supporting H17. The association of a woman’s satisfaction with food-related life and her own life satisfaction (spillover) was significantly higher than the association of a man’s satisfaction with food-related life and the woman’s life satisfaction (crossover) (*p* = 0.004), thus supporting H18.

In summary, as it was hypothesized, for men, spillover associations between job satisfaction and life satisfaction were significantly higher than crossover associations from women’s job satisfaction. Likewise, for women, spillover associations between satisfaction with family life and life satisfaction, as well as between satisfaction with food-related life and life satisfaction, were significantly higher than crossover associations from men’s family and food-related life satisfaction (Table 3). On the other hand, contrary to what was hypothesized, for men, the spillover association between satisfaction with family life and life satisfaction was significantly higher than the crossover association from women’s satisfaction with family life. For women, no statistical differences were found between spillover and crossover associations between their own and the men’s job satisfaction and their life satisfaction. Similarly, for men, no statistical differences were found between spillover and crossover associations between their own and the women’s satisfaction with food-related life and their own life satisfaction.

## 4. Discussion

Using the APIM approach, this is the first study that explores the spillover and crossover associations between job satisfaction, satisfaction with family life, satisfaction with food-related life and overall life satisfaction in dual-earner couples. As hypothesized, consistent with the bottom-up theoretical approach to life satisfaction [2,3], our results show positive relationships (or spillovers) between job satisfaction and life satisfaction in both members of the couple, which confirms previous studies conducted at an individual level in different countries [5,6,11,41,42,46,47,89]. Similarly, the positive spillover between satisfaction with family life and overall life satisfaction in both members of the dyad is consistent with previous studies conducted on the basis of the life satisfaction bottom-up approach, which concluded that the family domain is a significant contributor for life satisfaction at an individual level also in different countries [6,8,18,21,41,47,54]. Likewise, the findings supported a positive spillover between satisfaction with food-related life and overall life satisfaction in both members of the couple, in line with previous research examining the bottom-up approach to life satisfaction at an individual level [15,21,64,67]. 

Taking into account the contribution of the three domains to overall life satisfaction, the high strength correlation between satisfaction with family life and overall life satisfaction in both members of the couple is consistent with previous studies concluding that satisfaction in the family domain is a stronger contributor to overall life satisfaction than satisfaction in the job [8,19,42] and food [20,21] domains. The strong association between satisfaction with family life and overall life satisfaction is also in line with studies reporting that satisfaction with close relationships, such as a spouse, and relationships with one’s children made a greater contribution to overall life satisfaction than satisfaction in the job domain [4]. Nevertheless, it is worth noting that satisfaction with family life was the main contributor to life satisfaction for both members of the couple. This finding is in line with the results reported by Jovanović et al. [35] with a sample of undergraduate students in Iran regarding the lack of differences among women and men in the importance of social resources for overall life satisfaction. However, this result contradicts research conducted with undergraduate students in the US and Croatia indicating that social resources are more strongly associated with women’s life satisfaction than with men’s [35,68]. Thus, our results confirm that the importance of social resources, such as family life, for overall life satisfaction is age- and culture-sensitive [4,5,12], showing that the family domain is relevant for men and women in dual-earner couples in the stage of life under study (i.e., families with adolescent children), although the literature indicates that women get more involved in the family domain [26,74]. 

Regarding the relative importance of the two other domains as contributors to life satisfaction for women and men, the results support the suggestion that their different socialization practices and social roles may lead men and women to derive overall life satisfaction from different domains [35]. This can be seen in the results, as the spillover association between satisfaction with food-related life and life satisfaction was stronger than the association between job satisfaction and life satisfaction in women, while the opposite trend was found in men. As in other countries, in Chilean dual-headed households, women are still primarily responsible for feeding the family [56], while men are still considered the main provider [90]. In addition to gender roles and differences in socialization, there are other possible explanations related to the positive association between life satisfaction, satisfaction with food-related life and healthy diets [15,62,64,65,67]. The higher relevance of satisfaction in the food domain for women than for men may be related to the evidence showing that women have healthier diets than men [15,91], because women are more invested in engaging in healthful diets for their own and also for their families than men [38,56,92]. In addition, it has been found that male employees report more obstacles to achieving a high level of satisfaction with food-related life than women, such as lacking a fixed mealtime schedule and having insufficient time to eat at the workplace [37]. 

Between members of the couple, the following crossover effects were found: one symmetric or bidirectional positive crossover between satisfaction with family life and life satisfaction (from men to women and from women to men); one unidirectional or asymmetric positive crossover between job satisfaction and overall life satisfaction (from men to women). There were no crossovers between satisfaction with food-related life and overall life satisfaction. The findings regarding satisfaction with family life and job satisfaction are consistent with the suggestion by Liu and Cheung [25] that male partners may differ from their female partners in the significance or strength of the hypothesized crossover relationships, according to the type of outcome variables under consideration. Fredrickson’s “broaden and build theory” [93] may shed light on the underlying mechanisms through which one partner’s satisfaction with family life or job satisfaction affects the other partner’s life satisfaction. This theory suggests that positive emotions linked to an individual’s satisfaction in a determined domain promote externally oriented thoughts and actions; these responses further stimulate the person to respond positively to the needs of their partner by showing sympathy or concern about family or job issues, as in the present study. Accordingly, the partner perceives an improvement in the relationship, which in turn enhances their subjective well-being [25].

Consistent with the SCM [28], previous empirical findings [56] and authors who have reported symmetrical crossover associations among couples [31], our results show a significant correlation between one partner’s satisfaction with family life and the other partner’s life satisfaction, and vice versa. Namely, women’s satisfaction with family life crosses over to men’s life satisfaction, and men’s satisfaction with family life crosses over to women’s life satisfaction. These results are also in line with the Interdependence Theory [22] and the Family Systems Theory [23] regarding the interdependence between couples and members of a family. Although the spillover associations in each member of the couple were stronger than the crossover associations, these results show that an individual’s life satisfaction would not only be influenced by their own satisfaction with family life but also by their partner’s satisfaction with family life in dual-earner couples. 

The positive crossover between satisfaction with family life and overall life satisfaction between the couple members may be explained by the interrelations between both partners, who share significant aspects of their lives, especially in the family domain [30,32]. Therefore, in this context, it is likely that direct crossovers occur among members of a couple, meaning that partners transmit or exchange experiences, affective states and resources through empathy [94]. According to the mechanism of direct crossover, it can be suggested that satisfaction with family life in men produces an empathetic reaction in women that increases their life satisfaction, and vice versa [62]. However, it is worth highlighting that crossover was bidirectional. This finding may be related to an increase in positive interactions between partners when both are satisfied with their family life, which in turn increases their well-being [30]. However, further research is needed in more individualistic cultural contexts given the high importance of family in Latin American culture [95].

There was a unidirectional crossover from a man’s job satisfaction to his partner’s life satisfaction, whereas his life satisfaction was not associated with his partners’ job satisfaction. Following Westman [70], our findings show that a direct crossover occurs but only through women’s empathy, i.e., only men’s job satisfaction produces an empathetic reaction in women, which in turn increases their life satisfaction, but not vice versa. This result may be linked to a more traditional socialization of women [36], who are encouraged more than men to be attuned to the feelings and emotions of other people. In parallel, our findings are consistent with previous studies reporting that men tend to be less sensitive to their female partners’ positive experiences in the job domain, such as a female partner’s greater work-to-family enrichment and work-life balance [25,37]. These findings may also reflect traditional gender-based demands and expectations [70]. Given the traditional role of men as the family’s main “breadwinner” [74], as occurs in most of the sample under study, it is likely that the family’s financial situation depends more on the man’s than the woman’s job. Therefore, considering the importance of the financial situation to an individual’s life satisfaction [4,5,6,12,18,19], it is feasible that the greater importance of the man’s job for the family’s total income influence not only relates to their own overall life satisfaction but also to their partner’s. 

Contrary to what was expected [38], no crossover associations between satisfaction with food-related life and overall life satisfaction were found between partners. Other dyadic studies have reported a similar lack of crossover effects [30], yet this is an unexpected result in this study, as previous evidence suggests that individuals who share the same environment and experiences (i.e., family members) also share eating habits and satisfaction with food-related life [14,96]. This result may reflect a low frequency of shared meals in dual-earner couples [13,17,97], which is associated with time constraints, in particular for full-time employees [98] and also in dual-earner couples with conflicting work schedules [13]. Low family meal frequency has been related to unhealthy eating habits [13,97], as well as with lower levels of satisfaction with food-related life [14,62]. However, satisfaction with food-related life is about eating habits but also family interactions around mealtimes [14,21]. Research shows that family meals are an opportunity for family members to interact in a positive manner, providing emotional support and strengthening relationships with one another [60,61]. It can be thus hypothesized that when the frequency of family meals is low, as occurred in this sample, probably due to both partners having full-time jobs, one couple member’s satisfaction with food-related life does not cross over to the other members’ life satisfaction because they do not share their eating habits [14,96], nor do they experience the affective dimension of meals frequently [60,61]. However, further research is needed to explore the underlying causes of the lack of crossover in dual-earner couples, for example, comparing dual-earner couples with couples in which only one partner has paid employment, as well as including the work schedules of each partner.

Although most spillover associations were stronger than crossover associations in the relationship between the three domains of satisfaction and overall life satisfaction, the gender comparison suggests that gender differences are domain-dependent. While similar patterns for women and men were found in the relationship between satisfaction with family life and overall life satisfaction, different gender patterns may exist in the relationship between job satisfaction and overall life satisfaction as well as between satisfaction with food-related and life satisfaction in dual-earner couples. Therefore, our results are consistent with the findings reported by Yucel and Latshaw [73] in that gender differences in spillover and crossover relationships are associated with the variables under study. In the present study, the different gender patterns in the job and food domain are partially related to traditional gender roles, as we discuss below.

Our results show similar patterns for women and men in the relationship between satisfaction with family life and overall life satisfaction; i.e., although both couple members’ satisfaction with family life significantly crossed over to the other partner’s life satisfaction, the spillover association between each member of the dyad’s satisfaction with family life and life satisfaction was significantly higher than the crossover associations. This means that both partners’ life satisfaction is more susceptible to their own satisfaction with family life than to their partner’s. The higher spillover than crossover in women was an expected result, given that the primary domain for women is the home and family [26,70,74,98]. However, contrary to what was hypothesized, the same results were obtained for men. Although, as previously discussed, this result may be associated with Latin American culture [95], this finding may also reflect men being more actively engaged in family issues, as it has been reported in dual earner-couples in different countries [97,98]. This increases their involvement in the family domain, which in turn may lead to satisfaction with family life, a strong contributor to their overall life satisfaction. However, future research should test these findings in other Latin American countries. This is important, given that this finding may indicate a shift to more egalitarian gender roles, at least in the family domain in Latin American countries. 

In the job domain, while a man’s life satisfaction association with his own job satisfaction (spillover) was higher than the association with the woman’s job satisfaction (crossover), a woman’s life satisfaction was equally associated with her own job satisfaction (spillover) and her partner’s job satisfaction (crossover). This means that men’s life satisfaction is more susceptible to their own job satisfaction than their partner’s job satisfaction, whereas women’s life satisfaction is equally susceptible to their job satisfaction as well as their partner’s job satisfaction. These findings confirm the relevance of the work role for man’s identity [74], but it also expands the relevance of this role for both members of the couple. In fact, it has been reported that men’s main family role is being the breadwinner and their paid work is a way to prove their masculinity [99], more so in cultures with predominantly a traditional masculinity framing [100], as is the case in Latin American countries. 

By contrast, in the food domain, while the woman’s life satisfaction association with her own satisfaction with food-related life (spillover) was higher than the association with the man’s satisfaction with food-related life (crossover), men’s life satisfaction was equally associated with their own satisfaction with food-related life (spillover) and their female partner’s satisfaction with food-related life (crossover). Although for women and for men, crossover associations were non-statistically significant, the gender comparison indicates that men’s life satisfaction is equally susceptible to their own satisfaction with food-related life as well as to their female partner’s satisfaction with food-related life, whereas women’s life satisfaction is more susceptible to their own satisfaction with food-related life. This finding may be due to the gendered nature of food preparation. Despite advances towards gender equality in relation to food-related household work in countries around the world, the evidence shows that women invest more hours in the kitchen than men [101] and are the main ones responsible for the food-related tasks and family meals, even if they have paid employment [56]. Therefore, as satisfaction with food-related life also involves planning meals, shopping and preparing meals [58], it can be expected that if women can perform as well at work as in food-related tasks, their self-confidence may be reinforced, positively influencing both their satisfaction with food-related life and with their overall life. In this regard, a recent study stressed the importance for women of the availability and access to enough and healthy food to prepare family meals, which seems to be a key factor for women’s satisfaction with food-related life [37].

The limitations of this study should be acknowledged and corrected in future research. The first limitation is the study’s cross-sectional design, meaning that causal relationships between variables cannot be established. Longitudinal designs in future studies will allow to test for causality. The second limitation is that all data were self-reported and participants’ responses may have been affected by social desirability. A third limitation is the non-probabilistic sampling. This sample was representative of the socioeconomic status distribution in Chilean population [81] but it was not representative in terms of the participants’ age, number of family members or children per family. The average number of family members (four) and children (two) per family was higher than the average for Chilean families (three and one, respectively) [102]. Therefore, future studies should include representative samples in terms of Chilean family composition. Moreover, dual-earner couples in this study were parents of adolescent children, so the findings cannot be generalized to families at other stages of the life cycle. Related to the previous limitation, cross-cultural analyses are needed to include diverse Latin American countries, as well as countries with differences in family structure and gender equality, given that culture affects the contribution of life domains to overall life satisfaction [4,5,12] and the influence of gender roles [103]. Another limitation is that part of the crossover effects, and spillover effects that mirror those of the partner, may be explained by homogamy; that is, individuals’ tendency to become partners with or marry others with whom they share positive traits, such as happiness and life satisfaction [104]; further studies should address this potential confounding between transmission of experiences and shared traits. One last limitation is that we assessed the spillover and crossover associations only between three life domains and overall life satisfaction; therefore, future studies should include other important life domains, such as financial situation, health, leisure, living arrangements, friends and others. Future research should also explore possible moderators of these associations, not only in samples including dual-earner couples but also in parent–child dyads.

Despite these limitations, the results of the present study expand the understanding of how satisfaction in different life domain contributes to the individual’s overall life satisfaction by analyzing spillover and crossover associations in dual-earner couples on the basis of the SCM [28].

These results provide practical implications for dual-earner couples, policy makers and organizations. Although the spillover association between satisfaction with family life and life satisfaction was stronger than crossover associations, both couple members’ life satisfaction also increases if their partners are more satisfied with their family life. Therefore, both members of the couple should be concerned with the other partner’s satisfaction with family life and promote a healthy interaction between the two of them. At the same time, policy makers may develop strategies to enhance dual earner-couples’ satisfaction with family life, such as promoting flexibility and perceived control over one’s own schedule in employees [17], which would give both members of dual-earner couples enough time to fulfil their family’s responsibilities. Organizations should also seek to promote job satisfaction in employees of both genders, with special attention to male employees, given that their job satisfaction not only enhances their own life satisfaction but their female partner’s as well. 

Results from the present study also have important research implications. Studies on the relationship between domain satisfaction and overall life satisfaction have traditionally focused on individual household members. As most life domains involve activities and situations that take place in a social setting, this seems a limited scope. This study supports the use of APIM for modeling and analyzing such interdependencies, while showing that relationships between both members of dual-earner couples in life domains can help increase their life satisfaction. Thus, although dyadic sampling entails methodological and practical difficulties for researchers, more studies about the relationship between satisfaction in life domains and overall life satisfaction should consider the influence that individuals exert on others with whom they share a meaningful relationship.

## 5. Conclusions

Our results show that, via spillover, greater job satisfaction, satisfaction with family life and satisfaction with food-related life translate into higher levels of overall life satisfaction in both members of the dyad. Regarding crossovers, as it was hypothesized, a bidirectional crossover was found from one couple member’s satisfaction with family life to the other member’s life satisfaction. In the job domain, contrary to expectations, only a unidirectional crossover from men’s job satisfaction to their female partners’ life satisfaction was obtained, while in the food domains, no crossover associations were detected. Taken together, these findings suggest that family life has a more uniform influence on the life satisfaction of both men and women in a couple, followed by non-reciprocal associations from the job domain, and with food-related life limited to having an impact on life satisfaction at an individual level. Therefore, our results show that an individual’s life satisfaction may not only be influenced by their own satisfaction in a life domain, as the life satisfaction bottom-up approach poses [2,3], but it may also be influenced by their partner’s satisfaction in the same domain, in accordance with the Interdependence Theory [22] and the Family Systems Theory [23]. 

In addition, our results contribute to the knowledge about gender similarities and differences in the relationship between domain satisfaction and overall life satisfaction in dual-earner couples, showing, at the same time, different patterns in spillover and crossover associations in the job and the food domains. Some findings aligned with expectations, such as that men’s life satisfaction was associated with their own job satisfaction more than with their female partners’ job satisfaction; women’s life satisfaction was associated with their own family and food-related life satisfaction more than with those from their male partners. Other findings did not support the hypotheses, such as that men’s own family life was more strongly associated with their own life satisfaction (spillover) than women’s satisfaction with family life (crossover). Moreover, no statistical differences were found between spillover and crossover associations for job satisfaction in women and for satisfaction with food-related life in men. Because the contribution of life domains to overall life satisfaction is culture-sensitive [4,5,12], however, further research is needed in countries where the relationship between different-sex couples is more egalitarian.

## Figures and Tables

**Table 1:** Sample characteristics of participant couples (n = 473).

Characteristic	Total Sample	*p*-Value ^1^
Age (Mean (*SD*)) ^1^		
Woman	39.1 (7.2)	0.000
Man	42.0 (8.9)	
Number of family members (Mean (*SD*))	4.4 (1.0)	
Number of children (Mean (*SD*))	2.2 (0.8)	
Socioeconomic status (%)		
High	22.2	
Middle	61.5	
Low	16.3	
Gender of the main breadwinner (%)		
Female	23.3	
Male	76.7	
Number of days/week couples ate together (Mean (*SD*))		
Breakfast	2.8 (2.3)	
Lunch	3.3 (2.2)	
Dinner	2.5 (3.1)	
Satisfaction with life (SWLS) (Mean (*SD*)) ^1^		
Woman	23.2 (4.8)	0.003
Man	24.1 (4.6)	
Satisfaction with family life (SWFaL) (Mean (*SD*)) ^1^		
Woman	23.6 (4.8)	0.001
Man	24.7 (4.6)	
Satisfaction with food-related life (SWFoL) (Mean (*SD*)) ^1^		
Woman	21.3 (4.8)	0.001
Man	22.5 (4.6)	
Job satisfaction (OJSS) (Mean (*SD*)) ^1^		
Woman	22.3 (4.8)	0.540
Man	22.4 (5.0)	
Type of employment (%) ^2^		0.460
Woman employee	72.7	
Woman self-employed	27.3	
Man employee	74.8	
Man self-employed	25.2	
Working hours (%) ^2^		
Woman working 45 h per week	59.2	0.000
Woman working less than 45 h per week	40.8	
Man working 45 h per week	72.3	
Man working less than 45 h per week	27.7	

^1^ Independent sample t-test. ^2^ *p*-value corresponds to the (bilateral) asymptotic significance obtained in Pearson’s Chi-square Test.

**Table 2:** Factor loadings range, Omega coefficients, average variance extracted (AVE), correlations and squared correlations between the Overall Job Satisfaction Scale (OJSS), Satisfaction with Family life scale (SWFaL), Satisfaction with Food-related Life scale (SWFoL) and Satisfaction with Life Scale (SWLS) scores for each member of the couple.

Scale	Loadings Range (min–max)	Omega	AVE	Woman’s OJJS	Man’s OJJS	Woman’s SWFaL	Man’s SWFaL	Woman’s SWFoL	Man’s SWFoL	Woman’s SWLS	Man’s SWLS
Woman’s OJJS	0.518–0.911	0.91	0.65	-	0.110	0.071	0.063	0.077	0.038	0.114	0.057
Man’s OJJS	0.571–0.882	0.91	0.62	0.332 **	-	0.024	0.076	0.011	0.065	0.058	0.125
Woman’s SWFaL	0.771–0.934	0.94	0.75	0.266 **	0.155 **	-	0.250	0.140	0.135	0.549	0.205
Man’s SWFaL	0.716–0.955	0.92	0.71	0.250 **	0.275 **	0.500 **	-	0.078	0.226	0.239	0.587
Woman’s SWFoL	0.630–0.890	0.89	0.62	0.278 **	0.104 *	0.374 **	0.280 **	-	0.225	0.151	0.064
Man’s SWFoL	0.652–0.919	0.90	0.65	0.195 **	0.255 **	0.368 **	0.475 **	0.474 **	-	0.118	0.210
Woman’s SWLS	0.815–0.946	0.94	0.76	0.338 **	0.241 **	0.741 **	0.489 **	0.389 **	0.343 **	-	0.275
Man’s SWLS	0.749–0.843	0.93	0.71	0.239 **	0.354 **	0.453 **	0.766 **	0.253 **	0.458 **	0.524 **	-

The values over diagonal indicate squared correlations between constructs; The values under diagonal indicate correlations between constructs. * *p* ≤ 0.05; ** *p* ≤ 0.01

**Table 3:** Summary of hypotheses related to gender differences.

Hypothesis	Spillover	Relation Found	Crossover	Result
H13	Men’s job satisfaction to their own life satisfaction	>	Women’s job satisfaction to men’s life satisfaction.	Supported
H14	Women’s job satisfaction to their own life satisfaction	ns	Men’s job satisfaction to women’s life satisfaction.	Not Supported
H15	Men’s satisfaction with family life to their own life satisfaction	>	Women’s satisfaction with family life to men’s life satisfaction.	Not supported
H16	Women’s satisfaction with family life to their own life satisfaction	>	Men’s satisfaction with family life to women’s life satisfaction.	Supported
H17	Men’s satisfaction with food-related life to their own life satisfaction	ns	Women’s satisfaction with food-related life to men’s life satisfaction.	Not supported
H18	Women’s satisfaction with food-related life to their own life satisfaction	>	Men’s satisfaction with food-related life to women’s life satisfaction.	Supported

ns: not significant
